# Characterization of hormonal receptors and human epidermal growth factor receptor-2 in tissues of women with breast cancer at Muhimbili National Hospital, Dar es salaam, Tanzania

**DOI:** 10.1186/s13027-017-0170-5

**Published:** 2017-11-06

**Authors:** Amos Rodger Mwakigonja, Nyanda Elias Lushina, Ally Mwanga

**Affiliations:** 1 Department of Surgery, Muhimbili University of Health and Allied Sciences (MUHAS), Dar es Salaam, Tanzania; 2 Department of Pathology, Muhimbili University of Health and Allied Sciences (MUHAS), Dar es Salaam, Tanzania

**Keywords:** Breast cancer, Hormone receptors, ER/PR, HER2/neu, Tanzania

## Abstract

**Background:**

Breast cancer is a leading cause of morbidity and deaths among women worldwide. In Tanzania there is no published data on human epidermal growth receptor-2 (HER2/neu) expression in breast carcinoma. Hormonal receptors and HER2/neu status reportedly influence post-mastectomy adjuvant therapy and predict treatment outcome and prognosis. Here we evaluate hormonal receptors and HER-2 status in biopsies of women with breast cancer at Muhimbili National Hospital (MNH).

**Methods:**

A cross-sectional study of female breast post-modified radical mastectomy (MRM)/incisional biopsies confirmed to be carcinoma at the Histopathology Unit (January–December 2013). Tissue blocks having poor morphology, without tumor, secondary tumors, cases outside the study period and male patients were excluded. Routine staining was done followed by immunohistochemistry for estrogen (ER), and progesterone (PgR) receptors and HER2. Data analyzed using Statistical Package for Social Sciences (SPSS).

**Results:**

A total of 218 cases were confirmed to be carcinoma including 70 meeting inclusion criteria. Age at diagnosis ranged 18–75 years and mean age was 48.36 years. Majority (64.3%) were in the 36–55 years age-group. Histologically, most (88.6%) women had invasive ductal carcinoma including 43.1% of intermediate grade. A great majority (78%) were stage three. Due to logistical constrains, 75.7% (*n* = 53/70) cases where immunostained for hormones including 43.4% (ER+), 26.4% (PgR+), and 28% (ER+/PgR+). Furthermore, 65.7% (*n* = 46/70) cases were immunostained for HER-2 and 15.2% (*n* = 7/46) were positive, 45.6% were triple negative (ER-,PgR-,HER2-), 23.9% (ER+,PgR+,HER2-) or luminal B, 2.2% (ER+,PgR-,HER2+),13% (ER-,PgR-,HER2+) and 15% (ER+,PgR-,HER2-) with none being triple positive.

**Conclusions:**

Hormonal receptors and HER2 expression at MNH appears to be comparable to previous Africans/African Americans reports but not with studies among Caucasians and the current proportion of triple negative breast carcinomas (TNBC) is higher than in a previous Tanzanian report and majority are luminal. HER2 over-expression is relatively common. It is strongly recommended that receptor status assessment be made routine for breast cancer patients at MNH.

## Background

Breast cancer is the most common malignant tumour and leading cause of cancer deaths among women worldwide and the second most common malignancy among females in sub-Saharan African countries including Tanzania, after cancer of uterine cervix [1, 2]. More than half of the incident cases in the world occur in Europe and North America [3]. While data for developing countries is limited, cancer registries suggest that age-standardized incidence rates are rising even more rapidly in low-incidence regions such as Africa and Asia [4, 5]. Although reasons for these rising trends are not completely understood, but they may possibly reflect changes in reproductive patterns, obesity, physical inactivity and some breast cancer screening programs [6].

Breast cancer histogenesis is related to cells of either epithelial origin in tubules/ducts or mesenchymal cells. Extensive research suggests that genetic, hormonal and environmental factors may possibly be related to its aetiology [7, 8].

Prognostic factors are those which determine the outcome of disease in the absence of treatment whereas predictive factors predict response to treatment [9–11]. Thus, among other prognostic indicators for breast cancer, studies have shown that the presence of estrogen and progesterone receptors and HER-2 proteins to have influence in the prognosis of patients with breast cancer. [12] The presence of estrogen (ER) in particular as well as progesterone (PgR) receptors is important clinically, as a predictor of response to adjuvant hormonal therapy rather than prognostic factors [13, 14].

ER and PR are intracellular steroid hormone receptors which have received substantial attention since 1986. Measurable amounts of ER and PR are found in about 50–86% of patients with breast cancer [15].

Her2/neu gene amplification is another important prognostic and predictive factor for breast cancer. Approximately 20% of breast cancer patients have Her2/neu gene amplification which results in the glycoprotein overexpression. [16] This oncogene is associated with tumour aggressiveness and chemoresistance [17]. However, there are no data on the status of HER2 protein over-expression in breast cancers in Tanzania and our current study aims to elucidate the pattern of this expression for the first time in this country. Even in neighbouring Kenya ER/PR/HER2 receptor screening is not yet routine among breast cancer patients [18].

Furthermore, there have been different approaches in the management of breast cancer including screening at early stage, surgery and/or chemotherapy in combination with radiotherapy and hormonal therapy [19, 20].

Contrary to Europe and America, in Tanzania more than 90% of breast cancer patients present at an advanced disease stage [21]. This in part suggests that the biological behaviour of breast cancer in the Tanzanian population may be different besides delayed presentation to hospitals due to ignorance, poor access and/or initial consultation of traditional healers. In the past two decades, little has been reported about breast cancer in Tanzania except for a few studies of limited scale, all agreeing on the advanced disease stage at diagnosis [22]. Furthermore, a previous Tanzanian study from the regions around Lake Victoria has reported a low level of hormonal receptors with a significant (38.4%) proportion of Triple negative breast cancers although this has not yet been documented in other localities including in Dar es Salaam [23].

Thus our index study attempts to elucidate the pattern of ER, PgR and HER-2 expression as well as associated factors among women with breast cancer at MNH.

## Methods

### Study design

Hospital based analytical cross sectional study from January to December 2013.

### Study area

Muhimbili Nation Hospital (MNH) is a Public University Teaching Hospital and a National referral centre based in Dar es Salaam, the biggest commercial city in Tanzania. The hospital has a bed capacity of 1500 of which 120 beds are set apart for adult general surgery services. There are 64 female beds. Tanzania is an East African country about 5° south of the Equator with a population of about 45 million in 2012 (National Bureau of Statistics, March 2013). Tanzania is a relatively large country located in East Africa with a total area of 945,087 km^2^. Tanzania has 30 administrative Regions as well as about 128 districts. Dar es Salaam is the commercial/industrial capital with a population of about 4.4 million in 2012 according to National Bureau of Statistics, March 2013.

The standard of care for female patients with breast cancer is that as they attend clinics or when admitted FNAC is done to every patient with a breast lump, and for those with ulcerating tumour incision biopsy is done for Histopathology. Those with confirmed diagnosis for carcinoma of breast by histopathology are staged and managed according to their stage at presentation. From stage I-III, surgery is indicated followed by radio chemotherapy.

Surgical interventions include lumpectomy, simple mastectomy, radical or modified radical mastectomy (MRM) depending on stage. Most patients who are designated for surgery at MNH receive either MRM or simple mastectomy [24]. Breast tissue after mastectomy is taken for histopathology to access margins if free of tumour and also for confirmation of type of breast cancer before adjuvant chemoradiotherapy, hormonal and immunotherapy.

### Target population

All breast tissue specimens from women with confirmed diagnosis of breast carcinoma by histopathology attending the department of general surgery either as an outpatient or inpatient.

### Sampling (eligibility criteria)

**Inclusion criteria**; Breast tissue biopsy specimens of all stages at histopathology department from female patients self-presenting or referred to the department of general surgery with diagnosis of breast carcinoma confirmed by histopathology with good morphology and whose blocks are available in the archives during the study period.

**Exclusion criteria**;

i. Breast cancer in male patients
ii. Patients with benign conditions
iii. Secondary breast cancer arising from other sites
iv. Cases with missing tissue blocks and sections
v. Breast cancer diagnosed from cytological smears only

### Sampling

All breast tissue blocks with diagnosis confirmed for carcinoma at the histopathology laboratory were recruited into the study after being processed routinely for histopathological diagnosis. Then after collection of these tissue blocks a pathologist reviewed the slides to confirm the diagnosis and select blocks meeting criteria for immunohistochemical staining.

Then information on demographic characteristics, pathological stage and grade of tumour was retrieved and then entered into structured questionnaires.

### Tissue biopsies

Formalin-fixed and paraffin embedded (FFPE) tissue biopsy blocks were collected from the archives. Glass slides were stained with hematoxylin & eosin as previously described [25, 26]. The sections were reviewed to determine the histological type, stage, morphological state of the blocks and tumor grade, then immunostained for hormonal and HER2 protein over expression.

Questionnaires were filled there after by the researcher to obtain demographic data, type of breast cancer, pathological stage, grade, estrogen and progesterone status and HER2 status. A data sheet was also created to assist in keeping records.

### Data collection, handling and analysis

Data collected included investigation request forms, histopathology reports as well as clinical notes besides the slides and tissue blocks. Information was then entered into structured questionnaires were given serial numbers in addition to hospital numbers for systematic record keeping**.** Histopathology report copy for each tissue block was attached to respective questionnaire.

Data were then analyzed using the statistical programme for social scientists (SPSS) version 20. Pearson’s Chi-square and Fisher’s exact probability tests were used. A *P*-value of <0.05 was considered statistically significant.

### Specimen processing and immunohistochemical staining

Each tissue biopsy block from the archives was re-evaluated separately by one senior resident (Yahaya) and one pathologist (A. Mwakigonja) were H&E was done for histological diagnosis, type and tumor grade then pathological staging was done.

Haematoxylin and Eosin staining as previously described [25, 27].

#### Grading

The histological grading of invasive breast cancer was performed using the Elston-Ellis modification of Scarff-Bloom-Richardson system which scores the amount of glandular formation, nuclear pleomorphism and mitotic index of the tumor cells [28]. Each of these features were scored from 1 to 3, and then scores were added to get total scores which was ranging from 3 to 9 according to grading system. Total final score of 3–5 was graded as low grade (grade 1) and score of 6–7 was graded as intermediate grade (grade 2) and score of 8–9 was graded as high grade tumor (grade 3).

### Immunohistochemistry (IHC)

This was done of FFPE sections as previously described [25, 26]. The specimens for the immunoperoxidase staining procedure were retrieved from the formalin-fixed, paraffin wax-embedded tissue biopsies from the archives. A single representative 3 μm thick section for every tissue block was cut and then mounted on positively charged slides and then sections were deparaffinised by placing the slides on hot plate for 30 min at 60 °C and then dewaxed in 2 changes of xylene for a total of six minutes followed by rehydration through descending grades of alcohol and then stained as previously described [25, 27]. Primary antibodies from DAKO supplies Ltd. included hormonal receptors[ER (clone EP1) and ready to use dilution] and [PgR (clone 636) and ready to use dilution] and HER2 proteins [(clone Hercep test) and ready to use dilution] and incubated for 30 ± 1 min at room temperature. Both negative and positive controls were provided by Dako and also controls were available from routine staining protocols at MNH histopathology laboratory where negative controls for ER, PgR and HER2 included omitting the primary antibody.

### Microscopic evaluation criteria for ER/PR and HER2

After appropriate immunostaining was done and results for hormonal receptor expression were scored semi quantitatively by one senior Resident (Yahaya) and then reviewed by one experienced pathologist (A. Mwakigonja) using Reiner’s four-point scale based on intensity and percentage of IHC reaction [29]. For each antibody, microscopic evaluation of slides was done in which cell counting was performed separately to each section followed by recording the number and intensity of positive staining cells. A slide was considered positive when at least 10% of the cells had stained, with at least an intensity of 1+ to 4+. HER2 staining was evaluated according to manufacturer’s instructions (DAKO). Were grading for HER2 positivity was based on completeness of cytoplasmic membrane reactivity and percentage of tumor cells stained. Score of 0–1+ was considered negative for HER2 and score of 2+ was considered equivocal and 3+ or complete membrane staining was considered positive for HER2. [30] Tumor grading was based on nuclear pleomorphism, tumor differentiation (well differentiated, moderate and poorly differentiated) and mitotic figure counts per one high power field. Mitotic figure counts of 1–2 (low grade), 3–5 (intermediate grade) and above 5 (high grade).

## Results

### General results and demography

**Fig. 1:**
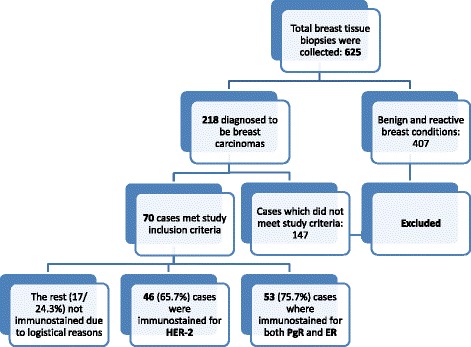
Flow chart showing how cases were collected for routine histopathology and immunohistochemical studies

**Fig. 2:**
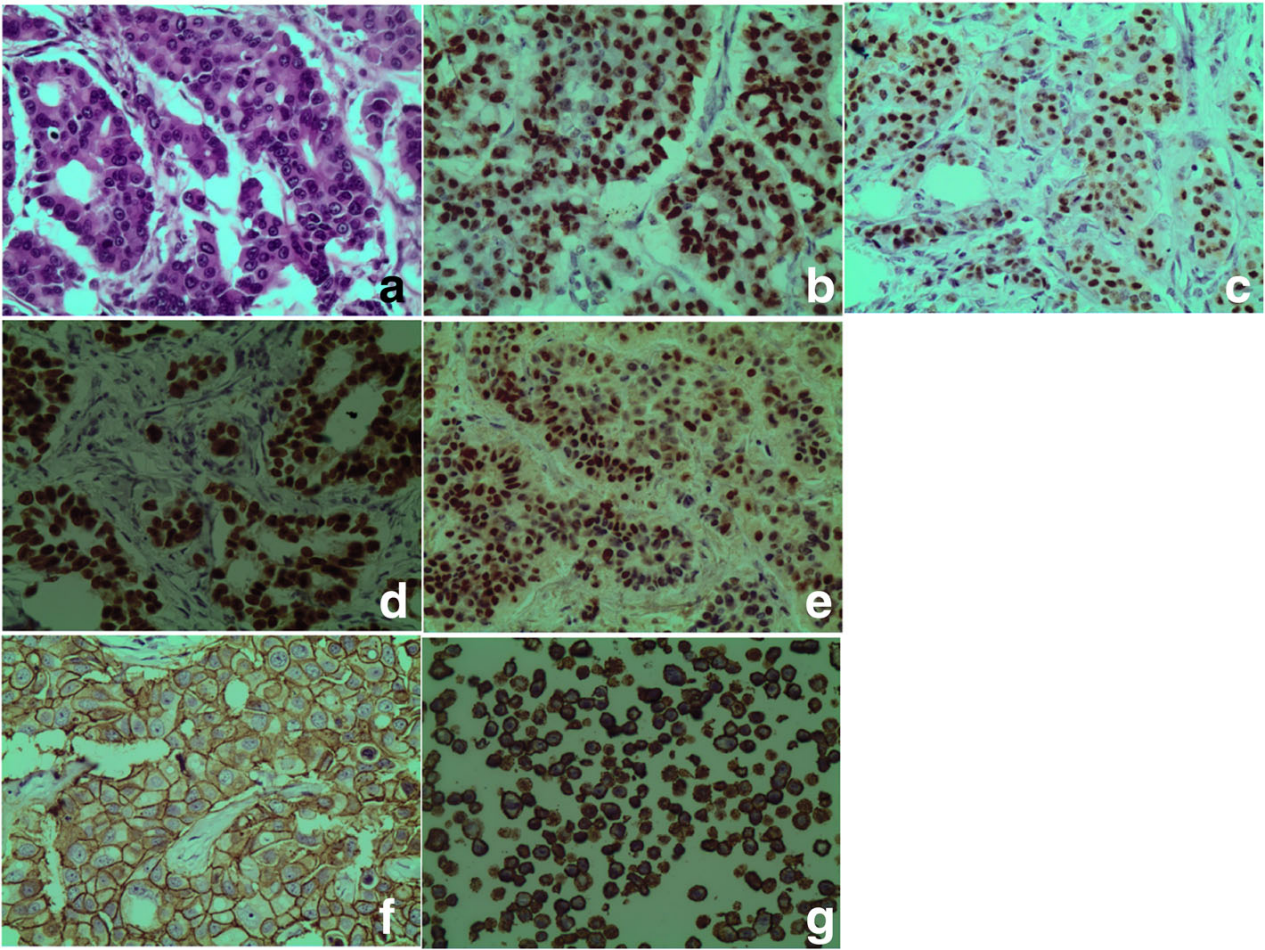
Micrographs: (**a**) H&E for invasive ductal carcinoma (IDC), (**b**) PgR 3+ immunoperoxidase nuclear staining in breast IDC, (**c**) PgR 3+ immunoperoxidase nuclear staining in a positive control tissue; (**d**) ER 2+ immunoperoxidase nuclear staining in breast IDC, (**e**) ER positive control; (**f**) HER-2 3+ immunoperoxidase cytoplasmic membranous staining, (**g**) HER-2 3+ positive control

Between the months of January and December 2013 a total of 625 breast tissue biopsies were collected from patients who attended MNH either as out-patients or as admitted including 218 confirmed to be carcinomas of which 70 cases met study inclusion criteria and thus stained by haematoxylin & eosin (H&E) [Figs. 1 and 2(a)]. Due to logistical constrains, only 53 (75.7%) cases where immunostained for both PgR [Figs. 1 and 2(b&c)] and ER [Figs. 1 and 2(d&e)]; while 46 (65.7%) cases were immunostained for HER-2 [Figs. 1 and 2(f&g)]. The age at diagnosis ranged from 18 to 75 years, with the mean being 48.36 ± 0.6 years. Peak age at diagnosis was between 36 and 55 years and accounted for 64.3% of study population (Table 1).

**Table 1:** Distribution of age-groups in the study population

Age groups at diagnosis (years)	Number of patients (%)
≤ 35	8(11.4)
36–55	45(64.3)^a^
> 55	17(24.3)
Mean age 48.36 ± 0.6	

^a^Peak age-group

### Clinico-pathological prognostic indicators of breast carcinoma

Only 54/70 women presented with a biopsy from the primary tumor and majority (48.1%, *n* = 26/54) of these had T4 disease followed by T3 disease (24.1%, *n* = 13/54) implying late presentation (Table 2).

**Table 2:** Prognostic indicators of breast carcinoma in study population other than Hormonal and HER2

Prognostic indicator	Number of patients (%)
Primary tumor	
Tx	2(3.7)
T1	2(3.7)
T2	11(20.4)
T3	13(24.1)
T4	26(48.1)
Total	54(100)
Histological grade	
High grade	24(36.9)
Intermediate grade	28(43.1)
Low grade	13(20.0)
Total	65(100)
Histological type	
Invasive ductal	62(88.6)
Invasive lobular	2 (2.9)
Medullary	3 (4.3)
^a^Others	3 (4.3)
Total	70(100)
Pathological stage	
Stage I	1(2)
Stage II	10(20)
Stage III	39(78)
Total	50(100)

^a^Other histological types included metaplastic and mucinous carcinomas

Regarding histological types of breast cancer among cases which met study inclusion criteria, infiltrating ductal carcinoma (IDC) was the most (88.6%, *n* = 62/70) common histological type of tumor seen, followed distantly (4.3%, *n* = 3/70) by medullary carcinoma while lobular carcinoma was the rarest (2.9%, *n* = 2/70) (Table 2). Other histological types included metaplastic carcinoma and mucinous type constituting 4.3% (*n* = 3/70) (Table 2). Histological grading was available for 65 cases (92.9%) and a great majority had intermediate-to-high grade (43.1%, 36.9% respectively or 80.0% together) implying advanced disease (Table 2). Furthermore, only 50/70 cases could be pathologically staged and again a great majority (78%, *n* = 39/50) of the patients had pathological stage III disease followed by 20% (*n* = 10/50) stage II also implying advanced disease (Table 2).

### Hormonal receptors and HER-2 expression

#### Estrogen and progesterone receptors

Out of 53 breast biopsies immunostained for estrogen and progesterone receptor status. 43.4% (*n* = 23/53) showed ER expression and the rest were negative (Table 3, Fig. 2 (d&e)). Furthermore, for PR status, a great majority 73.6% (*n* = 39/53) did not express the receptor except only 26.4% (*n* = 14/53) which expressed it (Table 4, Fig. 2 (b&c)) and 14 (26.4%) were positive for both estrogen and progesterone receptors expression which is also about 60.9% of those biopsies with estrogen receptor expression (Table 5). Moreover, all (100%) of estrogen receptors negative biopsies were also progesterone receptor negative (Pearson’s Chi square = 32.741, *P*-value < 0.0001), highly statistically significant (Table 5). It was also interesting to note that, progesterone receptor (PR) expression seemed to vary with pathological stage which finding was statistically significant (Pearson’s Chi square = 4.871, *P*-value = 0.027317, Fisher Exact Test) (Table 6). Conversely however, ER expression association with pathological stage was not statistically significant (*P*-value = 0.071944, Fisher Exact Test) (Table 6). The association of PR expression with primary tumor (T) size appeared not to be statistically significant (Pearson’s Chi square: 4.382, *P*-value = 0.11178, Fisher Exact Test) (Table 7) while ER expression was more (85.7%, *n* = 6/17) frequently associated with T2 tumors and this was statistically significant (Pearson’s Chi square: 5.846, P-value = 0.05, Fisher Exact Test) (Table 7).

**Table 3:** Frequency of estrogen receptors (ER) expression among studied biopsies at MNH

Estrogen Receptor (ER) Expression	Number of biopsies	Percent
Negative	30	56.6
Positive	23	43.4
Total	53	100.0

Majority (56.6%) of breast cancers were ER negative

**Table 4:** Frequency of progesterone receptors (PgR) expression in the studied biopsies

Progesterone Receptor (PgR) Expression	Number of biopsies	Percent
Negative	39	73.6
Positive	14	26.4
Total	53	100.0

A great majority (73.6%) of breast cancers were PR negative

**Table 5:** Frequency of ER/PgR co-expression in the studied biopsies

PROGESTERONE	ESTROGEN		Total
	Negative	Positive	
Negative	30(100%)	9(39.1%)	39(72%)
Positive	0(0%)	14(60.9%)	14(28%)
Pearson’s Chi square = 32.741 (*P*-value = 0.000)			
Total	30(100%)	23(100%)	53(100%)

About 26.4% (*n* = 14/53) of breast cancers co-expressed ER & PR

**Table 6:** ᅟ

Hormonal receptor & HER2 status	Pathological stage		
	Stage II	Stage III	Total
ER			
positive	5(29.4%)	12(70.6%)	17(100%)
Negative	1(5.9%)	16(94.1%)	17(100%)
0.071944	6	28	34(100%)
PR			
Positive	4(40.0%)	6(60.0%)	10(100%)
Negative	2(8.3%)	22(91.7%)	24(100%)
*P*-value 0.027317	6	28	34(100%)
HER2			
positive	0(0%)	3(100%)	3(100%)
Negative	6(20.7%)	23(79.3%)	29(100%)
*P*-value 0.249442	6	26	32(100%)

ER, PR & HER2 expression seemed more frequent in pathological stage III although this was statistically significant only for PR

**Table 7:** Relationship between hormonal receptor and HER-2 status expression with primary tumor size

Hormonal status &HER2	T2	T3	T4	Total
ER Positive	6(85.7%)	3(37.5%)	8(34.8%)	17(44.7%)
Negative	1(14.3%)	5(62%)	15(65.2%)	21(55.3%)
*P*-value 0.05	7(100%)	8(100%)	23(100%)	38(100%)
PgR Positive	4(42.9%)	2(25%)	4(17.4%)	10(26.3%)
Negative	3(57.1%)	6(75%)	19(82.6%)	28(73.7%)
*P*-value 0.138	7(100%)	8(100%)	23(100%)	38(100%)
HER2 Positive	1(14.3%)	5(55.6%)	1(5%)	7(19.4%)
Negative	6(85.7%)	4(44.4%)	19(95%)	29(80.6%)
*P*-value *P*-value = 0.00587	7(100%)	9(100%)	20(100%)	36(100%)

ER as well as PR expression seemed more frequent among those with primary tumor size T2 while HER2 was more frequent among T3

**Fig. 3:**
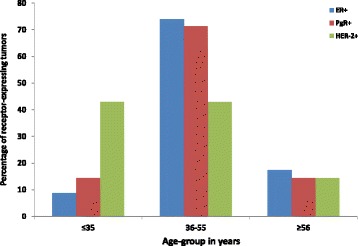
Histogram showing the percentage distribution of ER, PgR & HER-2 expression among biopsies of breast cancer women at MNH

Furthermore, our findings also suggest that, hormonal receptor expression has no statistically significant association with age group even though most (73.9%, *n* = 17/23) of the breast carcinoma with ER expression were seen in age group 36–55 years (Pearson’s Chi-square: 3.189, *P*-value = 0.203) and similarly (71.4%, *n* = 10/14) for PR expression Pearson’s Chi-square: 1.853, *P*-value = 0.396, Fisher Exact Test) (Table 8 and Fig. 3).

**Table 8:** Relationship between age as an independent prognostic factor with hormonal receptor and HER-2 status

Hormonal and HER2 status	AGE GROUP (YEARS)			
	≤35	36–55	≥56	Total
ER Positive Negative *p*-value 0.203	2(33.3%) 4(66.7%)	17(53.1%) 15(46.9%)	4(26.7%) 11(73.3%)	23(43.4%) 30(56.6%)
Total	6(100%)	32(100%)	15(100%)	53(100%)
PgR Positive Negative *P*-value 0.396	2(33.3%) 4(66.7%)	10(31.2) 22(68.8)	2(13.3%) 13(86.7%)	14(26.4%) 39(73.6%)
Total	6(100%)	32(100%)	15(100%)	53(100%)
HER2 Positive Negative *P*-value 0.01238	3(60%) 2(40%)	3(10.7%) 25(89.3%)	1(7.7%) 12(92.3%)	7(15.2%) 39(84.8%)
Total	5(100%)	28(100%)	13(100%)	46(100%)

#### HER2 expression

Our current study also found that only 15.2% IHC-tested breast cancer biopsies at MNH showed HER2 expression while the rest were negative for this protein (Table 9, Fig. 2 (f&g)) and although this was not statistically significant (*P*-value = 0.213), we found that out of 22 biopsies positive for estrogen receptors, only 4.5% were also positive for human epidermal growth factor receptors type-2 while the rest were negative including 76% which were negative for both HER2 and ER (Table 10). Furthermore, although it was not statistically significant, it was still interesting to note that there was no mutual PR and HER2 co-expression in the tumours of our cohort (P-value = 0.171) (Table 11).

**Table 9:** Frequency of HER-2 expression in the studied biopsies

HER-2 Status	Number of biopsies	Percent (%)
Negative	39	84.8
Positive	7	15.2
Total	46	100

Only 15.2% of those tested were positive for HER2

**Table 10:** Frequency of ER/HER2 co-expression in the studied biopsies at MNH

HER-2 Status	ESTROGEN		Total
	Negative	Positive	
Negative	18(76%)	21(95.5%)	39(84.8%)
Positive	6(24%)	1(4.5%)	7(15.2%)
Total	24(100%)	22(100%)	46(100%)

ER/HER2 co-expression was minimal (4.5%)

**Table 11:** Frequency of PgR/HER2 co-expression in the studied biopsies at MNH

	PROGESTRONE Receptor		Total
	Negative	Positive	
HER-2 Status			
Negative	28(77.8%)	11(100%)	39(84.8%)
Positive	7(12.2%)	0(0%)	7(15.2%)
Total	35(100%)	11(100%)	46(100%)

In contrast to both ER and PR expression above, HER2 expression was the same for age groups of **≤**35 years and 36–55 years respectively and this was highly statistically significant (Pearson’s Chi-square: 8.783, *P*-value = 0.01238, Fisher Exact Test) [Fig. 3].

Regarding primary tumor size (T), majority (55.6%) of women with T3 tumors expressed HER2 and this finding was highly statistically significant (Pearson’s Chi square: 10.276, *P*-value = 0.00587, Fisher Exact Test) (Table 7). Furthermore, there was no statistically significant relationship between HER2 expression with pathological stage (Pearson’s Chi-square = 1.326, *P*-value = 0.249442, Fisher Exact Test) (Table 6) and the relationship between hormonal status and HER2 with histological type of breast cancer was not statistically significant mostly due very small numbers of lobular, medullary and other non-ductal carcinomas (data not shown).

#### Double and triple receptor expression

**Fig. 4:**
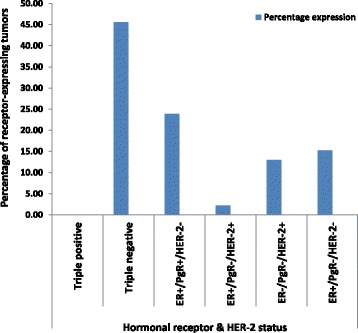
Histogram showing the percentage distribution of ER, PgR & HER-2 expression by histological grade among biopsies of breast cancer women at MNH

Regarding combined hormonal and HER2 expression, we found no triple positive tumors at MNH during the study period. Triple negative breast carcinomas (TNBC) were 45.6% (*n* = 21/46), tumors with ER+/PR+/HER2– (Luminal) were 23.9% (*n* = 11/46), and only one case (2.2%) expressed both ER and HER2 but negative for PR (Table 12, Fig. 4). HER2+ tumors (also called Her2-Enriched) with negative expression of both ER and PR were 13% (*n* = 6/46) and those tumors expressing ER with negative PR and HER2 were 15.2% (*n* = 7/46) [Fig. 4].

**Table 12:** Proportion of ER, PgR and HER2/ in the studied biopsies at MNH

Hormonal status	Disease Subtype	No. (%)	
Triple positive		0(0%)	
Triple negative		21(45.6%)	
ER+/PR+/HER2-	Luminal^a^	11(23.9%)	
ER+/PR−/HER2+	Luminal-HER2 enriched/positive (Luminal B)	1(2.2%)	
ER−/PR−/HER2+	HER2 enriched/positive	6(13%)	
ER+/PR−/HER2-	Unclassified	7(15.2%)	
Total		46(100%)	

^a^This could not be subclassified into Luminal A or B since Ki-67 could not be done to ascertain the histological grade due to logistical constrains

**Fig. 5:**
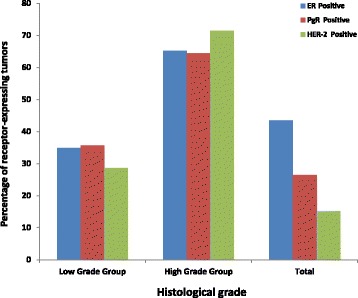
Histogram showing the percentage ER, PR & HER-2 expression with age-groups of women with breast cancer at MNH

Furthermore, in the current study we regarded intermediate- and high-grade breast cancers as being of higher grade in contrast to those that were low-grade since they are both more likely to be more biologically aggressive than the later group. Thus, majority (65.2%, 64.3% and 79.5% respectively) of ER+, PR+ and HER2 positive tumors respectively were found in the intermediate to high grade group and. This association appeared to be statistically significant with a P-value of 0.041 implying that, tumor grade might influence both hormonal as well as HER2 receptor expression (Fig. 5).

## Discussion

The protocol of clinical management of breast carcinoma patients at MNH for many years has been surgery (mastectomy) followed by chemo-radiotherapy, hormonal therapy using Tamoxifen and recently immunotherapy using Trastuzumab (Herceptin) at the Ocean Road Cancer Institute (ORCI) [24].

All patients with breast cancer attending ocean road until recent years were given hormonal therapy (Tamoxifen) irrespective of their menstrual status; [31] and often without being evaluated for PR and ER protein expression by immunohistochemistry as recommended [32]. Although it is recommended that all patients with breast cancer newly diagnosed must have HER2 test performed, patients at MNH-ORCI have generally not had the access to these tests [33].

The present study comprised of 70 female breast tissue biopsies confirmed to be carcinoma of which 53(75.7%) cases were immunostained for both ER and PR and this sample size does not differ much from the study done twelve years ago in Tanzania were 60 patients were included in the study. [34] However, in the current study immunostaining for HER2 could be done for only 46 cases although to the best of our knowledge, this was the first time that marker has been documented from Dar es Salaam, Tanzania.

The mean age at diagnosis was 48.36 years with age range between 18 and 75 years with most of the patients having age between 36 and 55 years. This age-group at presentation that we report in our current study, is generally in agreement with a previous Tanzanian study were the mean age at diagnosis was 43.4 years [20]. In that previous study, it was also found that 11.4% of cases had age below 35 years. Young age at diagnosis is reported in different literatures to be associated with aggressive disease, human epidermal growth factor receptor-2 positivity and poor prognosis. In Nigeria 28.96% of patients with breast cancer were diagnosed at age below 40 years of which, 42% were <35 years [35, 36].

Furthermore, although data was not shown, a significant (48.1%) proportion of patients assessed for primary tumors had infiltration of the skin and the chest wall implying that they were advanced. Moreover, it was also interesting to note that only 3.7% of patients with primary tumors had masses less than 2 cm in size and 24.1% were larger than5cm. This presentation of patients with large tumors seem to have persisted during the last decade as one can also be seen from a previous study done on surgical specimens from breast cancer patients at MNH were the mean tumor size was 8 cm, and none had presented with tumor size less than 20 mm and moreover majority (68.8%) were tumors exceeding 5 cm maximum diameters [34].

Most of the patients reviewed for histological grade of tumors in this study had intermediate and high grade. These findings differ from a previous Irish report were 53% of invasive primary breast cancer had low to intermediate grades and that high grade tumors were only 47%. [37] The difference in grades at presentation may be explained by delayed presentation resulting in larger tumor sizes among Tanzanian breast cancer patients compared to those in Western countries [38].

As expected, in the current study, majority (88.6%, *n* = 65) patients reviewed for histological type of tumor, (had invasive ductal carcinoma which concurs with different previous reports from different countries [39, 40].

Furthermore, our current study shows that 75% out of 51 patients reviewed were in pathological stage 3 and this was similar to the findings previously reported from Tanzania which showed that 90.7% of the study population were in stage 3 and 4 and concluded that majority of the Tanzanian breast cancer women present at a late stage [22].

In the present study, estrogen receptor positive tumors were 43.4% out of 53 breast tissue biopsies immunostained for estrogen receptor and that majority (56.6%) of the tested samples did not express receptors for estrogen. These findings corroborate those reported previously by a Tanzanian study involving 60 patients immunostained for hormonal receptors at MNH and found that estrogen receptor expression among female breast cancer patients was 33% implying that majority of patients here are estrogen receptors negative [34].

Similarly, another study done at ORCI and MNH five years ago on clinical and epidemiological profile of breast cancer in Tanzania, showed that out of 57 tumors immunostained for hormonal status 49.1% were ER−/PR- [22]. Different studies in Nigeria have consistently reported that hormonal receptor negative tumors are predominant among West African women with breast cancer with only about 24% and 25% ER positive in 178 and 124 cases respectively [41, 42].

Estrogen receptors expression in African women living in America and in Britain is consistently reported to be between 61% and 66% [40, 43]. In the present study we have seen that ER expression was 10 % more common than in previous studies in Tanzania among women with breast cancer [34]. This difference could partly be due to sample size and time differences including selection bias.

In the current study expression of progesterone receptors was 26.4% with majority (73.6%) of these being negative for progesterone receptors expression. Progesterone receptors expression in breast tissue biopsies has been reported to range from 13.9% to 61.3% of both primary and metastatic breast tumours [42, 44]. A previous Tanzanian report showed that only 18% out of 60 studied cases of breast cancer were progesterone receptor positive [34]. One study in Cuba in 1982 produced the first report on hormonal receptors expression of women with breast cancer using biochemical methods, showing that 56% were ER positive and 43% PR positive [45].

The two Tanzanian findings are not in agreement with the Cuban findings partly due geographical, racial, ethnic as well as sample size differences. It is noteworthy however, that PR expression seems to be increasing in Tanzanian samples at MNH. As regards hormonal receptors co-expression, our current findings that only 26.4% showed positivity while the rest were negative compare well with those reported from Sudan and Nigeria but not with a previous Tanzanian study on the clinical and epidemiological profiles of 57 breast cancer patients which found that 41% had ER/PR co-expression [22, 41, 46]. The differences between the two Tanzanian studies could partly be due to sampling biases, differences in the assays techniques and timeframes.

The present study has found that majority (84.8%) of breast tissue biopsies immunostained for HER2 protein did not express this receptor, with only about 15.2% of these being positive. Because this is the first study to be done in Tanzania on HER2 protein expression, there is no data available locally for comparison although, reports from elsewhere show that the expression of HER2 protein occurs in 13–20% of invasive ductal breast carcinoma and more than half (55%) of these cases were found to be hormonal receptor negative [47].

Studies have found that breast cancers expressing HER2 protein are aggressive and have poor prognosis in the absence of immunotherapy drugs including Trastuzumab [48].

Furthermore, an Australia study involving different methods of HER2 testing found the expression of HER-2 protein to be 14.6% while different studies in India have reported HER-2 positivity to be 17% and 26% respectively [49, 50]. These results are generally in agreement with our findings of 15.2% implying possible similarities in HER-2 protein expression among breast cancer patients from these different populations. We note however, that HER2 expression in Tanzanian patients is slightly on the lower side implying less likelihood of our patients to benefit from related interventions like Herceptin although, the differences could also be partly due to poor tissue handling, improper fixation and processing time and improvised fixatives. This low receptor expression even for ER and PR has also been reported from Mwanza, Tanzania previously [23]. As proposed by ASCO/CAP HER2 guideline which says that the fixative should be 10% neutral well-buffered formalin and fixation time should be within 6–8 h and not more than 72 h before processing and that slicing of breast tissue biopsies should be at 2-5 mm interval and placed into formalin [32]. All these SOPs may not be fully in practice in our settings by both surgeons and pathologists thus leading to current findings of lower expression. It is noteworthy that HER2 results have direct implication on patient management.

It was found by our current study that about 45.6% of studied female breast cancers were triple negative (ER−/PR−/HER2-) or TNBC which is comparable but higher than in a previous Tanzanian report (38.4%); and well in concordance with the incidence of47% in African American women in contrast to the 22% in white women [23, 51]. Furthermore, it was also found in the current study that luminal (ER+/PR+/HER2-) were 23% (this was not sub-classified into A or B because Ki-67 immunostaining was not performed due to logistical constrains) and luminal B (ER+/PR−/HER2+) [also called luminal-HER2 group] were 2.2% and HER2+/ER−/PR- (HER2 positive or HER2-enriched group) subtype were 13% and unclassified subtypes (ER+/PR−/HER2-) were 15.2%. Our results are in agreement with those from six geographical regions in Nigeria and Senegal on population differences in breast cancer which similarly reported that luminal A were 27%, luminal B were (2%) and HER2+/ER- were 15% and triple negative were 27% and unclassified were 28% [52]. Furthermore, these studies findings indicate we could have found a similar distribution pattern of luminal A and B had we also done Ki-67 since these are similar sub-Saharan African populations. Thus, the current study showed that most of the breast cancer patients were triple negative (TNBC) and these are grouped as poor prognosis which is in concordance with a previous Tanzanian report from Bugando, Mwanza. [23] Furthermore, the finding of 15.2% unclassified ER+/PR−/HER2- tumors was interesting and novel in Tanzania and will help allow comparative research and interventions with other countries [9, 34, 53].

The present study has found that HER2 receptor expression or positivity was 85.7% (*n* = 6/7) in estrogen negative and only 14.3% (*n* = 1/7) in positive tumors. Furthermore, that all (100%) positive HER2 over-expressers were progesterone negative. This apparently, shows an inversely proportional relationship between hormone receptor expression, with that of HER2. These inverse findings imply that we may expect an increased prevalence of HER2 expression in Tanzania and sub-Saharan Africa in general when breast cancer immunostaining becomes routine because of the prevalent hormonal receptor negativity.

Our finding that most (80%) of the estrogen receptor positive tumours were low grade underlines the known fact that hormone receptor positivity is associated with better prognosis, [12] and this finding was statistically significant. Similarly, a study in India showed that both progesterone and estrogen receptor expression were significantly related to low grade tumors [54].

The present study also found that HER2 positivity had no significant relationship with tumor grade. Similar findings were also seen in a previous report were HER2 over expression was significantly related to grade in Caucasians but not in African Americans. [53] These findings are in agreement with those in a previous report from America where HER2 expression was also more frequent among young African American women but not in Caucasian counterparts underlining the ethnic/racial similarities in biological behaviour of breast cancer among people of African descent [55].

Furthermore, we report that majority (60%) of patients expressing HER2 protein belonged to the younger age-group (<35 years) and this was statistically significant. Conversely, with advanced age, the number of patients negative for HER2 expression increased.

The current study found that majority (66.7%) of patients with progesterone receptor positivity were in stage two disease and this was statistically significant and these findings were not consistent with a previous report from Iran were progesterone positivity was found more frequently in patients of higher disease stage [56]. Reasons for this disparity are unclear but could in part be due to racial, ethnic, geographical and sampling differences. However, the association of ER and HER2 expression with pathological stage was not statistically significant**.**

Furthermore, in the present study, estrogen receptor expression appeared to be most (85.7%) frequent in primary tumor size T2 (2-5 cm) and this was statistically significant. This appears to agree with previous reports from elsewhere [9, 57]. Furthermore, the present study found that late-stage presentation of patients at MNH with medium tumor size is common and was associated with ER expression thus giving hope for improved survival when breast cancer immunohistochemistry is routine and hormonal therapy is given. Conversely, the findings of our index study shows that HER2 expression was statistically significantly associated with larger (>5 cm) primary tumor sizes. This seems to be in agreement with findings from a previous Italian study [58]. However, HER2 positive tumors may be amenable to Herceptin.

### Limitations for the study

This study did not cover the index of aggressiveness for breast cancer (ki67) due to logistical constrains thus limiting further sub-classification of some subtypes.

Another limitation was the lack of adequate retrievable clinical information.

## Conclusions

Hormonal receptors and HER2 protein expression in association with various biological characteristics of female breast patients attending MNH is generally comparable to those of other indigenous Africans with breast cancer as well as with African Americans but does not appear to compare well with expression among Caucasians. Majority of our patients at MNH are triple negative with good number in Luminal as well as in the unclassified groups. Furthermore, a good number of our patients are HER2 over-expressers and so are grouped as poor prognosis in the absence of adjuvant immunotherapy. Age and primary tumor size were found to influence HER2 but not hormonal receptor expression and there seems to be an inverse association between HER2 over-expression and hormonal receptor status in patients attending MNH. It is recommended that hormonal receptors as well as HER2 and Ki-67 status assessment be made routine to all patients with breast cancer attending MNH to allow prognostication and guide treatment.

## Abbreviations

ER: estrogen receptor
HER-2 or HER2 or HER2/neu: human epidermal growth factor receptor-2
MNH: Muhimbili National Hospital
ORCI: Ocean Road Cancer Institute
PR or PgR: progesterone receptor
TNM: Tumor, Node (lymphnode), Metastasis (distant metastasis)
